# Supplementary material for: Content-rich biological network constructed by mining PubMed abstracts
Source: BMC Bioinformatics. 2004 Oct 8;5:147. doi: 10.1186/1471-2105-5-147 (PMC528731; doi:10.1186/1471-2105-5-147)
Supplement: Additional File 2 — The original results of the above study (non-essential files are deleted to keep the file size under the limit set by BMC bioinformatics). [file 1471-2105-5-147-S2.bz2 › chilibotAdditionalFile2/dip20/539ID9211894E10892/html/JUN_AR.html]

 


 **JUN** and **AR** 
  
Found 81 abstracts in PubMed, retrieved 20.  
 

 What does Google say? 
 PDF only 
| .edu only 

---

**Interactive relationship** (e.g. stimulation, inhibition, etc)

**Inhibitory relationship**- These results suggest that AhR can stimulate AP 1  [ **JUN** ]  expression resulting in inhibition of the binding of  **AR**  to ARE in the transcription regulatory region of target genes such as PSA.  Ref: 12799773 Arch Toxicol, 2003
- Inhibition of  **AR**  also attenuated the increase in p38 mitogen activated protein kinase and c  **Jun**  N terminal kinase phosphorylation.  Ref: 12490536 FASEB J, 2003
**Neutral relationship**- In neonatal cardiomyocytes, activation of the G coupled alpha adrenergic receptor  **AR**  induces hypertrophy by activating mitogen activated protein kinases, including c  **Jun**  NH terminal kinase.  Ref: 12433842 Circ Res, 2002
- The  **AR**  acetylation site point mutants showed wild type trans repression of NF kappa B, AP 1  [ **JUN** ] , and Sp1 activity.  Ref: 11971970 Mol Cell Biol, 2002

**Non-interactive relationship** (e.g. studied together, co-existance, homology, etc.)

- Our results show that curcumin down regulates transactivation and expression of  **AR** , activator protein 1  [ **JUN** ] , nuclear factor kappaB, and CREB binding protein.  Ref: 12239622 Int J Oncol, 2002
- Since activator protein 1  [ **JUN** ]  AP 1  [ **JUN** ] , a heterodimer of c  **jun**  and c fos proteins, is known to inhibit binding of  **AR**  to ARE by protein protein interaction with  **AR** , the findings in the present study suggest a possible involvement of AP 1  [ **JUN** ]  in the antiandrogenic effects of PAHs acting as AhR agonists.  Ref: 12799773 Arch Toxicol, 2003
- Furthermore, recombinant PIASxalpha enhances Ubc9 mediated sumoylation of the androgen receptor  [ **AR** ]  and c  **Jun**  in vitro.  Ref: 12077349 Mol Cell Biol, 2002
- We report that, identically to 17beta estradiol or dihydrotestosterone, but differently from raloxifene, estren alters the activity of Elk 1, CCAAT enhancer binding protein beta C EBPbeta, and cyclic adenosine monophosphate response element binding protein CREB, or c  **Jun**  c Fos by an extranuclear action of the ER or  **AR** , resulting in activation of the Src Shc ERK pathway or downregulation of JNK, respectively.  Ref: 12782668 J Clin Invest, 2003
- In our previous study, we found a new mixed formula of Chinese herbs containing Shin yi san Xiao qing long tang Xiang sha liu  **jun**  zi tang 9 3 3 g divided in three doses day was beneficial to the patients with perennial allergic rhinitis  **AR**  via complicated immunomodulatory effects on both mononuclear cells MNC and polymorphonuclear neutrophils PMN.  Ref: 12469937 Int Immunopharmacol, 2002
- Agonist activation of the alpha  **AR**  resulted in increased p38 kinase activity, but not c  **Jun**  NH terminal kinase activity.  Ref: 11752101 J Pharmacol Exp Ther, 2002
- ... inhibition of c Fos, c  **Jun** , HB EGF,  **AR** , and VEGF mRNA by this compound was incomplete 41 65%.  Ref: 12016209 J Biol Chem, 2002
